# Primary PEComa of the bladder treated with primary excision and adjuvant interferon-alpha immunotherapy: a case report

**DOI:** 10.1186/1471-2490-6-20

**Published:** 2006-08-22

**Authors:** Jeremy R Parfitt, Anthony J Bella, Bret M Wehrli, Jonathan I Izawa

**Affiliations:** 1 Department of Pathology, London Health Sciences Centre and University of Western Ontario, London, Canada; 2 Department of Surgery, Division of Urology, London Health Sciences Centre and University of Western Ontario, London, Canada

## Abstract

**Background:**

Perivascular epithelioid cell tumors (PEComas) are rare mesenchymal neoplasms of uncertain malignant potential, which have in common the co-expression of muscle and melanocytic immunohistochemical markers.

**Case presentation:**

A 48-year-old man presented with dysuria, passage of urinary sediment and lower abdominal discomfort. A three centimeter mass was identified by cystoscopy in the posterior midline of the bladder. Computerized tomography suggested an enterovesical fistula. The patient underwent laparotomy, partial cystectomy and partial small bowel resection. Pathological examination revealed PEComa of the bladder. The patient underwent adjuvant interferon-α immunotherapy. Subsequent follow-up procedures, including cystoscopy and imaging, have not revealed evidence of recurrence. The patient is clinically free of disease 48 months after surgery.

**Conclusion:**

This case represents the second documented PEComa of bladder and demonstrates that adjuvant therapies, including anti-angiogenic and immunotherapy, may be considered for patients with locally advanced or metastatic genitourinary PEComa.

## Background

Primary perivascular epithelioid cell tumors (PEComas) are a rare and unusual group of mesenchymal neoplasms with unpredictable malignant potential. The term "PEComa" was originally coined by Zamboni et al and is the current nomenclature for tumors composed of PECs other than angiomyolipoma (AML), clear cell sugar tumor of lung (CCST) and lymphangioleiomyomatosis (LAM), which are related lesions with distinct clinical features [1]. Consequent to the World Health Organization's (WHO) endorsement of PEComa as a bonafide entity, an increasing number of reports have documented PEComas arising in varied anatomic locations, including bladder, kidney and prostate [2-8]. Despite increasing awareness of this entity, accurately predicting the biological behavior of PEComas remains difficult and contemporary reports are limited by short clinical follow-up. Herein, we report the diagnosis, management and four-year follow-up of the second documented case of primary PEComa of the urinary bladder [8].

## Case presentation

A 48-year-old man presented with dysuria, passage of urinary sediment and lower abdominal discomfort. A three-centimeter (cm) smooth, lobular mass with mild bullous edema was identified by cystoscopy in the posterior midline of the bladder. Laboratory and staging investigations were negative and computerized tomography (CT) suggested an enterovesical fistula. The patient underwent laparotomy, partial cystectomy and partial small bowel resection. Pathological examination revealed PEComa of the bladder. A search for primary melanoma was negative and there were no stigmata of tuberous sclerosis. The patient underwent adjuvant interferon (IFN)-α immunotherapy for primary PEComa of the bladder. Subsequent follow-up evaluations were performed 3 monthly for the first 12 months, then 6 monthly thereafter. Routine follow-up procedures included clinical examination, cystoscopy, chest roentgenography, CT of abdomen and pelvis and routine blood work. On one occasion, positron emission tomography was performed in order to detect neoplastic metabolic activity. None of these follow-up procedures revealed evidence of recurrence. The patient is clinically free of disease 48 months after surgery.

## Pathology

**Figure 1:**
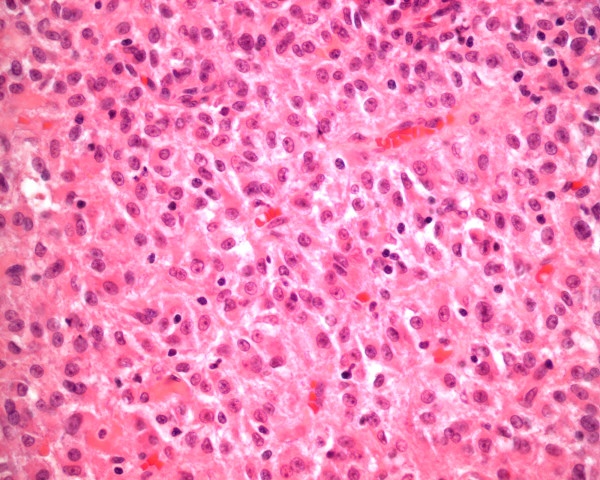
Tumor cells were mainly epithelioid with abundant eosinophilic and granular cytoplasm. Occasional nuclear inclusions were present (hematoxylin-eosin, original magnification ×400).

A fragmented specimen, 11 cm in aggregate, containing pieces of friable, tan-colored tumor, as well as bladder and small bowel tissue, was received for pathological examination. Histologically, the tumor was located in the bladder wall, but also showed infiltration into subserosal, muscularis propria and submucosal layers of the small bowel. The neoplastic cells were mainly epithelioid but occasionally spindled, with abundant cytoplasm that varied from eosinophilic and granular to clear (Figure 1). The nuclei were round with little pleomorphism; they often contained discernible nucleoli and occasional nuclear inclusions were present. Mitoses were rare to absent and necrosis was inconspicuous. There was no evidence of fat or thick-walled vessels. Periodic acid-Schiff staining, with and without diastase digestion, confirmed the presence of abundant intracytoplasmic glycogen.

**Figure 2:**
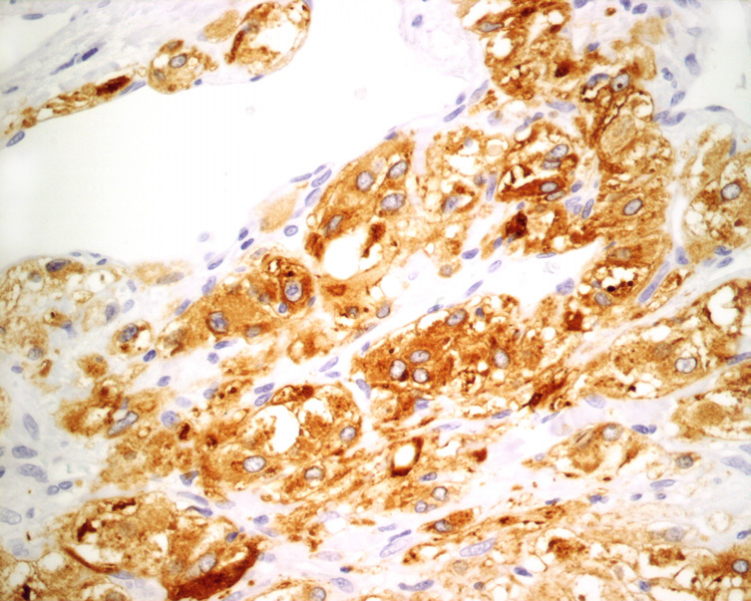
Tumor cells demonstrated strong and diffuse cytoplasmic positivity for HMB45 and Melan-A (Melan-A immunoperoxidase, original magnification ×400).

**Figure 3:**
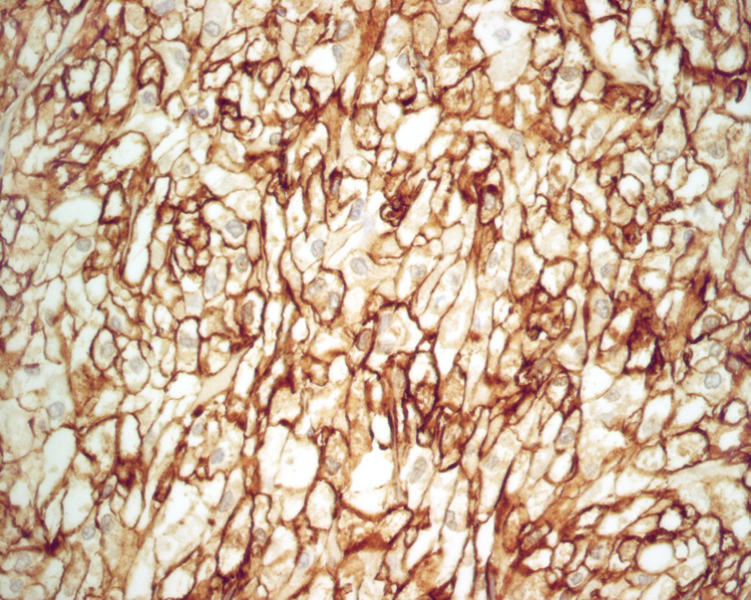
Tumor cells showed strong and diffuse membranous positivity for smooth muscle actin (smooth muscle actin immunoperoxidase, original magnification ×400).

Immunohistochemically, >80% of the tumor cells demonstrated strong positivity for HMB45 (cytoplasmic), Melan-A (cytoplasmic) and smooth muscle actin (membranous), while smooth muscle myosin heavy chain, desmin and CD117 were weakly positive in <20% of the tumor cells (Figures 2 &3). The neoplastic cells failed to stain with antibodies against S100 protein, cytokeratin (AE1/AE3, 8/18), vimentin, muscle specific actin, myoglobin, CD31, CD34 and WT-1. Positive controls were used for all markers as follows: HMB45 and Melan-A – melanoma; S100 – schwannoma; desmin, smooth muscle actin, muscle specific actin, smooth muscle myosin heavy chain and myoglobin – gastrointestinal (GI) smooth muscle; CD117 – GI mast cells and Cajal cells; cytokeratin – GI epithelia; vimentin – GI mesenchyma; CD34 and CD31 – tonsillar endothelia; WT-1 – renal glomeruli. Slides stained omitting the primary antibody were used as negative controls.

## Discussion

The WHO has offered formal recognition to a group of neoplasms with perivascular epithelioid cell differentiation [2]. These tumors have in common the presence of epithelioid to spindle cells with eosinophilic to clear cytoplasm that, with few exceptions, demonstrate positive immunostaining for markers of both myoid (smooth muscle actin, desmin) and melanocytic (HMB45, Melan-A, tyrosinase) differentiation. Pathologically, the main differential diagnoses include paraganglioma, melanoma, clear cell sarcoma of soft parts (CCSSP), metastatic carcinoma (especially from kidney or adrenal gland) and epithelioid sarcoma.

While distinct clinicopathological entities included within the PEComa group include AML, LAM and CCST, other PEC-derived tumors have been documented at an increasing number of anatomical sites, including pancreas, small and large intestine, ligamentum teres/falciform ligament, common bile duct, bladder, prostate, breast, uterus, cervix, vulva, ovary, broad ligament, heart, base of skull, and soft tissue [3-8]. The term "PEComa" has become the popular umbrella term for this latter list of lesions. Only one case of primary bladder PEComa has been described previous to ours. Pan et al reported a case of PEComa occurring in the deep detrusor muscle of the bladder in a 33-year-old woman, which was an incidental discovery during work-up for dysmenorrhea [8]. While there were no worrisome clinicopathological features of the tumor and the patient remained tumor free during the entire six-year follow-up period, there was no description of post-operative treatment.

PEComas demonstrate uncertain tumor biology and unpredictable clinical behavior. While the majority of reported "PEComas" have behaved in a benign fashion, an important minority have demonstrated malignant behavior with locally destructive recurrences, distant metastases and patient death, underscoring the need for accurate identification and effective treatment strategies [4,5,7]. When Folpe et al combined results of 24 of their own cases of PEComa of soft tissue and gynecological origin with data from 45 previously reported cases of PEComa, they found that recurrence and metastasis were associated with tumor size >5 cm, infiltrative growth pattern, high nuclear grade, necrosis and a mitotic index of >1 per 50 high power fields [9,10]. However, other authors feel that accurate criteria which reliably predict the behavior of PEComas remain lacking [10]. In the present case, the surgical margins were not evaluable, due to the fragmented nature of the specimen, and lymph nodes were not sampled, since the intraoperative impression was that of a benign enterovesical fistula, rather than a potentially malignant neoplasm.

Optimal treatment for PEComas is not known at this time. Primary excision is usually curative, as most tumors are benign. However, locally advanced or metastatic disease portends a poor prognosis and strategies incorporating chemotherapy, radiation and immunotherapy have been reported. In this patient, a one-year course of adjuvant IFN-α 2b at 10 million units given subcutaneously three times per week was initiated based on the vascular nature of this tumor and IFN-α's additional anti-angiogenic effect [11]. While IFN-α 2b therapy for the management of PEComa remains experimental, other authors have described the efficacy of IFN-α 2a in causing regression of life-threating hemangiomas in infants [12,13]. While fever, neutropenia and skin necrosis have been reported as uncommon, short-term side effects of IFN-α 2a, no such effects were seen in the present case [12]. As there was no evidence of residual tumor in the present case, a limitation of our report would be that the effect of IFN-α on PEComa morphology could not be documented. Thus, further studies are needed to clarify the clinical and pathological effects of IFN-α therapy in patients with PEComa and the risks of IFN therapy should be weighed against the potential benefits in any patient lacking detectable residual tumor. Partial, complete and absent responses have also been noted for dacarbazine, vincristine and imatinib mesylate, a tyrosine-kinase inhibitor [14]. Adjuvant radiation for CCSSP has also been reported following wide surgical excision, with primary site irradiation appearing to confer a survival benefit for disease located in soft tissue of the extremities [15].

## Conclusion

In summary, we report the first case of PEComa of the bladder treated with adjuvant IFN-α immunotherapy, with long-term follow-up. Given the uncertainty of PEComa tumor biology, adjuvant therapies, including anti-angiogenic and immunotherapy, may be considered for patients with locally advanced or metastatic genitourinary PEComa.

## Competing interests

The author(s) declare that they have no competing interests.

## Authors' contributions

JRP prepared the manuscript.

AJB helped to provide patient history and helped in the writing of the manuscript.

BMW was involved with diagnosis of the pathologic specimen and contributed to the writing of the manuscript.

JII performed the surgery, provided patient history, obtained patient consent and contributed to the writing of the manuscript.

All authors read and approved the final manuscript.

## Pre-publication history

The pre-publication history for this paper can be accessed here:
